# Natural Convection of Ternary Hybrid Nanofluid in a Differential-Heated Enclosure with Non-Uniform Heating Wall

**DOI:** 10.3390/mi14051049

**Published:** 2023-05-14

**Authors:** Vemula Rajesh, Mikhail Sheremet

**Affiliations:** 1 Department of Mathematics, GITAM (Deemed to be University), Hyderabad Campus, Hyderabad 502329, Telangana, India; v.rajesh.30@gmail.com; 2 Laboratory on Convective Heat and Mass Transfer, Tomsk State University, 634050 Tomsk, Russia

**Keywords:** square cavity, free convection, ternary hybrid nanofluid, linear heating side wall, numerical simulation

## Abstract

In the field of convective energy transfer, natural convection is one of the most studied phenomena, with applications ranging from heat exchangers and geothermal energy systems to hybrid nanofluids. The aim of this paper is to scrutinize the free convection of a ternary hybrid nanosuspension (Al_2_O_3_-Ag-CuO/water ternary hybrid nanofluid) in an enclosure with a linearly warming side border. The ternary hybrid nanosuspension motion and energy transfer have been modelled by partial differential equations (PDEs) with appropriate boundary conditions by the single-phase nanofluid model with the Boussinesq approximation. The finite element approach is applied to resolve the control PDEs after transforming them into a dimensionless view. The impact of significant characteristics such as the nanoparticles’ volume fraction, Rayleigh number, and linearly heating temperature constant on the flow and thermal patterns combined with the Nusselt number has been investigated and analyzed using streamlines, isotherms, and other suitable patterns. The performed analysis has shown that the addition of a third kind of nanomaterial allows for intensifying the energy transport within the closed cavity. The transition between uniform heating to non-uniform heating of the left vertical wall characterizes the heat transfer degradation due to a reduction of the heat energy output from this heated wall.

## 1. Introduction

During the last several years, there has been a lot of focus on the occurrence of natural convection in enclosures. This phenomenon has attracted attention primarily because it frequently affects thermal performance in a wide range of fundamental and industrial applications, including those involving heat exchangers, chemical reactors, solar collectors, fire systems, and the electronics, chemical, and power energy apparatus. Several thermal engineering applications use natural convection to remove heat without the aid of outside motion. Many heat transfer applications, including solar collectors, electronic device cooling, heat exchangers, and energy storage tanks, among others, make use of the benefits of natural convection [1,2,3,4,5].

Thermal systems’ performance and compactness are primarily constrained by the weak heat conductivity of common energy transport liquids like ethylene-glycol (EG) or water. In recent years, a novel method for enhancing heat transmission that uses nano-sized additives located in a host liquid, known as nanosuspension, has undergone substantial research [6,7,8]. It is also significant to highlight that new kinds of nanofluids, referred to as hybrid nanosuspension, can be created using improved attributes. Different nano additives are disseminated in a host liquid to create hybrid nanosuspension. By balancing the benefits and drawbacks of individual nanoparticles, hybrid nanofluids can produce designed liquids with modified thermal and chemical attributes [9,10,11].

The slow heat transmission between the fluid and the walls is one of natural convection’s key drawbacks. As a result, strategies for increasing the rate of heat transfer have been developed. These approaches include creating cavities with complicated geometries, employing cavities filled with porous media, adding fins to the wall(s), using magnetic fields or using nano and hybrid nanofluids. The thermal convection of a hybrid Al_2_O_3_-Cu/H_2_O nanosuspension was studied by Mehryan et al. [12] in a heated porous enclosure. An entropy generation study was provided by Tayebi and Chamkha [13] for a hybrid nanofluid moving in an MHD thermal convection motion via an enclosure with a corrugated conducting block. The conjugate thermal convective motion of a Ag-MgO/H_2_O nanosuspension was investigated by Ghalambaz et al. [14] in an enclosure. In a hybrid nanosuspension area, Chamkha et al. [15] investigated the MHD thermogravitational energy transfer of a localised heater/cooler. In a chamber saturated with a hybrid nanosuspension and a solid cylinder, Tayebi and Chamkha [16] analysed the entropy generated as an outcome of MHD thermal convective flow. Free convection and entropy generation were investigated by Tayebi et al. [17] in a hybrid nanosuspension saturated-elliptical chamber that generates or absorbs heat from within. Using an irregular solid circular cylinder, Tayebi and Chamkha [18] examined the MHD thermal convective energy transport of a hybrid nanosuspension in a chamber. Nanofluid natural convection in a square cavity subjected to thermal radiation was investigated by Reddy and Sreedevi [19] based on a model developed by Buongiorno. In a region with an elliptical barrier, Belhaj and Ben-Beya [20] reported a thermal investigation of thermal convection using hybrid nanofluids and a varying magnetic field. Nabwey et al. [21] used a hybrid nanofluid with a square obstruction to investigate the radiative influence on transient MHD thermal convection circulation in an inclined irregular porous chamber.

In addition, ternary hybrid nanofluids have lately become the focus of study to further accelerate the rate of heat transmission. For a porous prismatic chamber with two moving heated obstacles, Shao et al. [22] investigated the natural convection of ternary hybrid nanofluids. Employing changeable diffusion and a non-Fourier’s notion, Algehyne et al. [23] established a computational approach to ternary hybrid nanofluid flow. Using ternary-hybrid nanofluids, Elnaqeeb et al. [24] studied the effects of suction and dual-stretching on the three-dimensional motion of water-carrying nano additives of varying geometries and densities. Numerical simulations of ternary nanosuspension circulation with different slip and heat jump restrictions were published by Alshahrani et al. [25]. Convective energy transport in a ternary nanofluid that is moving over a stretching plate was theoretically explored by Manjunatha et al. [26]. Other related recent works can be found in [27,28,29,30,31,32,33,34,35,36,37,38,39,40,41].

In light of the numerous applications in engineering and technology, as well as the literature mentioned above, in this paper, the thermal convection of a ternary hybrid nanosuspension (Al_2_O_3_-Ag-CuO/water) in an enclosure is investigated. Linearly heating the side wall is considered. The PDEs controlling the liquid circulation and energy transport with suitable boundary conditions are described by using the single-phase nanofluid model. The finite element technique based on the COMSOL Multiphysics simulation software is applied to resolve the control PDEs after transforming them into a dimensionless form. The effect of governing characteristics, such as the nanoparticles, concentration, Rayleigh number, and linearly heating temperature constant on the velocity and temperature fields using streamlines and isotherms and mean Nusselt number at the heated border with appropriate patterns, has been investigated and analyzed.

## 2. Mathematical Analysis

**Figure 1:**
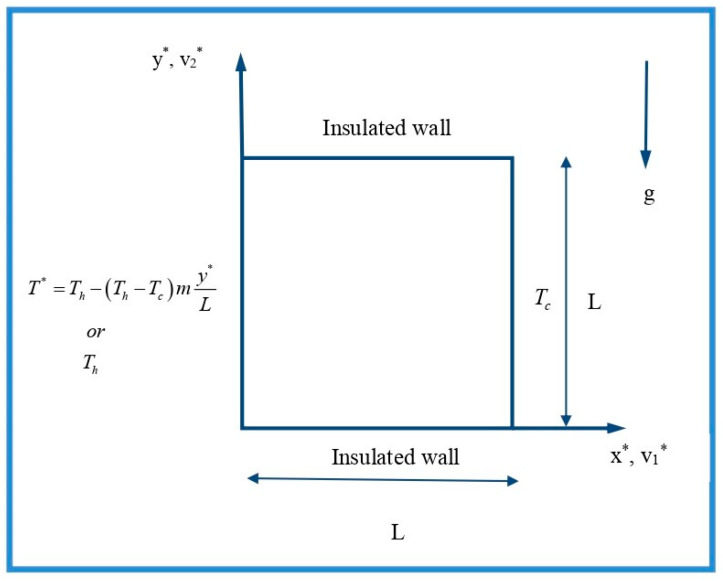
Problem sketch and system of coordinates.

We consider a square enclosure of size *L* for the analysis, as presented in Figure 1. The vertical border of the left side is linearly warmed, with a temperature pattern of $T^{*}\left(0,y^{*}\right)=T_{h}-\left(T_{h}-T_{c}\right)\;m\;\frac{y^{*}}{L}$, and the right vertical border is cooled with a temperature of $T^{*}\left(L,y^{*}\right)=T_{c}$. The other two (horizontal) walls are well-insulated. For this investigation, ternary hybrid nanofluid Al_2_O_3_-Ag-CuO/water is considered as the working fluid. In addition, there is no inner thermal production, and the present research disregards the effects of radiation and viscous dissipation. The operating fluid is a Newtonian fluid that is incompressible and has constant characteristics. The Oberbeck–Boussinesq equations have been applied to model the issue for 2D steady and laminar circulation situations. Changes in the density of the nanofluid are taken into account using the Boussinesq approximation. The host liquid (water) and the nano additives are believed to be in heat equilibrium. Below is a representation of the continuity, momentum, and energy equations: [42,43]

(1) $$\frac{\partial v_{1}^{*}}{\partial x^{*}}+\frac{\partial v_{2}^{*}}{\partial y^{*}}=0$$

(2) $$\rho_{thnf}\left(v_{1}^{*}\frac{\partial v_{1}^{*}}{\partial x^{*}}+v_{2}^{*}\frac{\partial v_{1}^{*}}{\partial y^{*}}\right)=-\frac{\partial p^{*}}{\partial x^{*}}+\mu_{thnf}\left(\frac{\partial^{2}v_{1}^{*}}{\partial x^{*}{}^{2}}+\frac{\partial^{2}v_{1}^{*}}{\partial y^{*}{}^{2}}\right)$$

(3) $$\rho_{thnf}\left(v_{1}^{*}\frac{\partial v_{2}^{*}}{\partial x^{*}}+v_{2}^{*}\frac{\partial v_{2}^{*}}{\partial y^{*}}\right)=-\frac{\partial p^{*}}{\partial y^{*}}+\mu_{thnf}\left(\frac{\partial^{2}v_{2}^{*}}{\partial x^{*}{}^{2}}+\frac{\partial^{2}v_{2}^{*}}{\partial y^{*}{}^{2}}\right)+{\left(\rho \beta \right)}_{thnf}g\left(T^{*}-T_{c}^{*}\right)$$

(4) $$v_{1}^{*}\frac{\partial T^{*}}{\partial x^{*}}+v_{2}^{*}\frac{\partial T^{*}}{\partial y^{*}}=\frac{\kappa_{thnf}}{{\left(\rho c_{p}\right)}_{thnf}}\left(\frac{\partial^{2}T^{*}}{\partial x^{*}{}^{2}}+\frac{\partial^{2}T^{*}}{\partial y^{*}{}^{2}}\right)$$

The additional border restrictions are

(5) $$\begin{array}{l} v_{1}^{*}\left(x^{*},0\right)=v_{1}^{*}\left(x^{*},L\right)=v_{1}^{*}\left(0,y^{*}\right)=v_{1}^{*}\left(L,y^{*}\right)=0,\\ v_{2}^{*}\left(x^{*},0\right)=v_{2}^{*}\left(x^{*},L\right)=v_{2}^{*}\left(0,y^{*}\right)=v_{2}^{*}\left(L,y^{*}\right)=0,\\ \frac{\partial T^{*}}{\partial y^{*}}\left(x^{*},0\right)=\frac{\partial T^{*}}{\partial y^{*}}\left(x^{*},L\right)=0,\\ T^{*}\left(0,y^{*}\right)=T_{h}-\left(T_{h}-T_{c}\right)\;m\;\frac{y^{*}}{L},\\ T^{*}\left(L,y^{*}\right)=T_{c} \end{array}$$

The physical attributes of ternary nanosuspension, including density ρ*_thnf_*, viscosity μ*_thnf_*, thermal volume capacity (ρ*c_p_*)*_thnf_*, volume heat expansion parameter (ρβ)*_thnf_*, and heat conductivity κ*_thnf,_* are shown in Table 1 and Table 2.

Utilizing the below-stated transformations

(6) $$x=\frac{x^{*}}{L},\;y=\frac{y^{*}}{L},\;v_{1}=\frac{v_{1}^{*}L}{\alpha_{f}},\;v_{2}=\frac{v_{2}^{*}L}{\alpha_{f}},\;T=\frac{T^{*}-T_{c}}{T_{h}-T_{c}},\;p=\frac{p^{*}L^{2}}{\rho_{f}\;\alpha_{f}^{2}}$$

in the above Equations (1)–(4), we get

(7) $$\frac{\partial v_{1}}{\partial x}+\frac{\partial v_{2}}{\partial y}=0$$

(8) $$\frac{\rho_{thnf}}{\rho_{f}}\left(v_{1}\frac{\partial v_{1}}{\partial x}+v_{2}\frac{\partial v_{1}}{\partial y}\right)=-\frac{\partial p}{\partial x}+Pr\frac{\mu_{thnf}}{\mu_{f}}\left(\frac{\partial^{2}v_{1}}{\partial x^{2}}+\frac{\partial^{2}v_{1}}{\partial y^{2}}\right)$$

(9) $$\frac{\rho_{thnf}}{\rho_{f}}\left(v_{1}\frac{\partial v_{2}}{\partial x}+v_{2}\frac{\partial v_{2}}{\partial y}\right)=-\frac{\partial p}{\partial y}+Pr\frac{\mu_{thnf}}{\mu_{f}}\left(\frac{\partial^{2}v_{2}}{\partial x^{2}}+\frac{\partial^{2}v_{2}}{\partial y^{2}}\right)+\;Ra\;\;Pr\frac{{\left(\rho \beta \right)}_{thnf}}{{\left(\rho \beta \right)}_{f}}\;T$$

(10) $$\frac{{\left(\rho c_{p}\right)}_{thnf}}{{\left(\rho c_{p}\right)}_{f}}\left(v_{1}\frac{\partial T}{\partial x}+v_{2}\frac{\partial T}{\partial y}\right)=\frac{\kappa_{thnf}}{\kappa_{f}}\left(\frac{\partial^{2}T}{\partial x^{2}}+\frac{\partial^{2}T}{\partial y^{2}}\right)$$

The corresponding boundary conditions are

(11) $$\begin{array}{l} v_{1}\left(x,0\right)=v_{1}\left(x,1\right)=v_{1}\left(0,y\right)=v_{1}\left(1,y\right)=0,\\ v_{2}\left(x,0\right)=v_{2}\left(x,1\right)=v_{2}\left(0,y\right)=v_{2}\left(1,y\right)=0,\\ \frac{\partial T}{\partial y}\left(x,0\right)=\frac{\partial T}{\partial y}\left(x,1\right)=0,\\ T\left(0,y\right)=1-\;m\;y,\\ T\left(1,y\right)=0 \end{array}$$

Here, $Pr=\frac{\upsilon_{f}}{\alpha_{f}}$ is the Prandtl number, and $Ra=\frac{g\beta_{f}\left(T_{h}-T_{c}\right)L^{3}}{\upsilon_{f}\;\alpha_{f}}$ is the Rayleigh number.

The quantity of practical interest in this research is the average Nusselt number, which is given as $Nu_{Average}=-\frac{\kappa_{thnf}}{\kappa_{f}}\;\int_{0}^{1}{\left(\frac{\partial T}{\partial x}\right)}_{x=0}dy$.

## 3. Numerical Solution

The non-dimensional Equations (7)–(10) and the boundary conditions provided in (11) are solved using the COMSOL Multiphysics simulation software, adopting the Galerkin finite element technique. To achieve optimum balance between numerical accuracy and associated computational cost, the best mesh type is determined by examining a number of elements that range from extremely coarse to extremely fine with the average Nusselt number. Finally, a 16,946-element extra fine mesh is selected, which has shown to be satisfactory due to an insignificant change in the average Nusselt number. This grid refinement study shown in Table 3 confirms that the mesh statistics provided in Table 4 are found to be optimal in the context of balancing accuracy and computational time.

**Figure 2:**
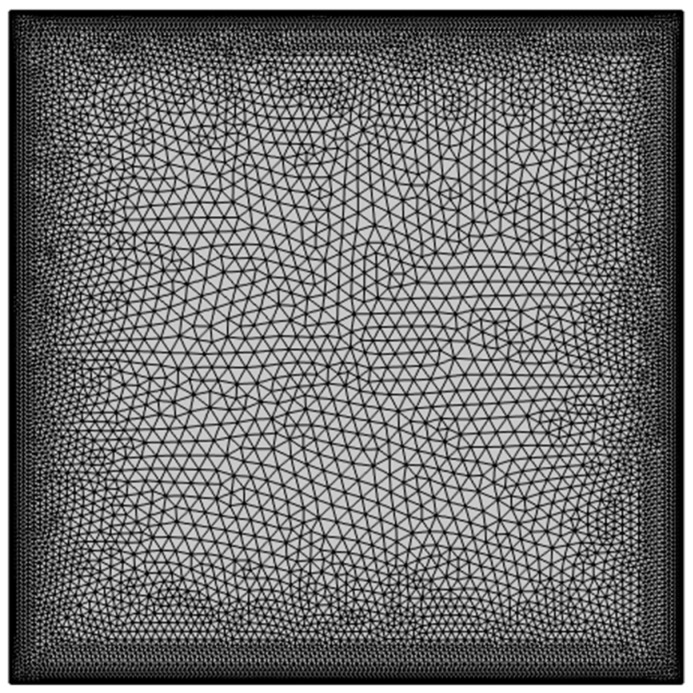
Computational grid chosen after mesh sensitivity analysis.

**Figure 3:**
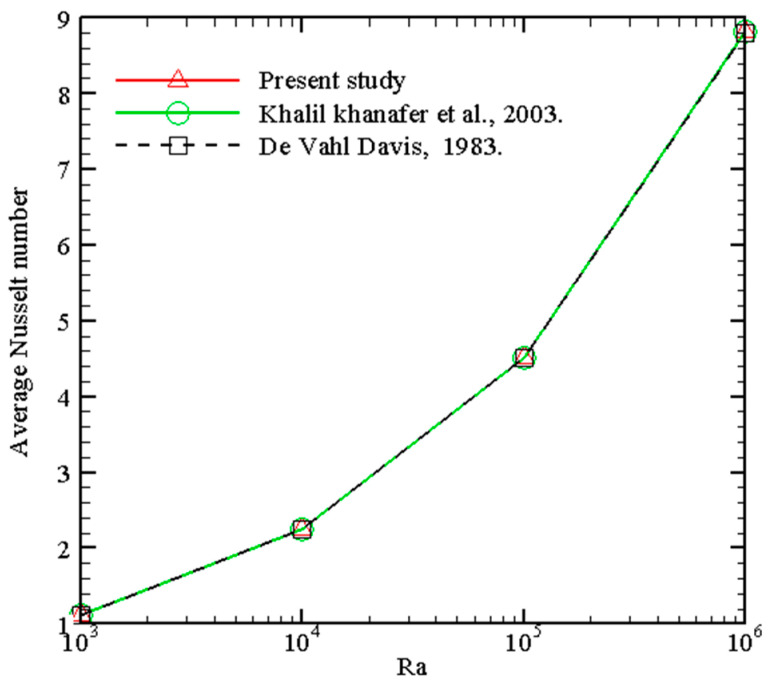
Comparison of Nusselt numbers. Red line: Present study; Green line: Khalil Khanafer et al. 2003 [45]; Black line: De Vahl Davis, 1983 [46].

**Figure 4:**
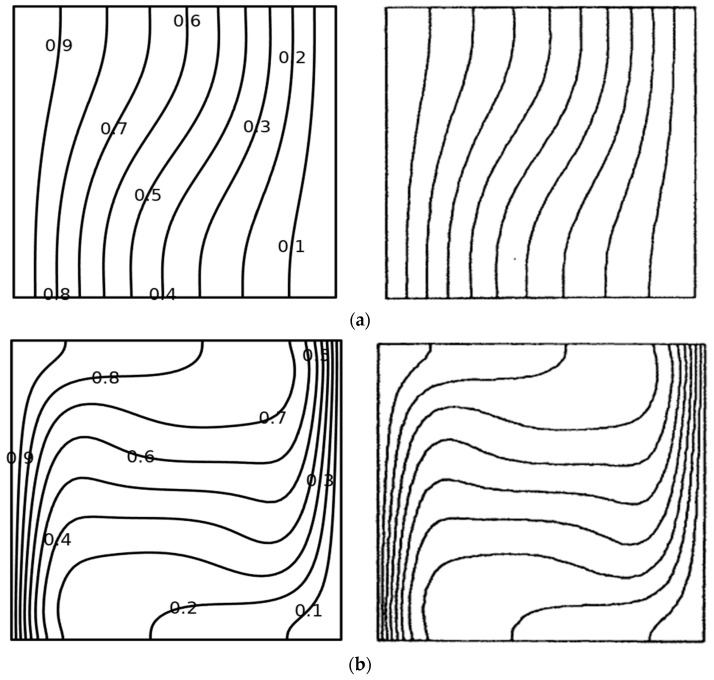
Comparison of isotherms. (**a**) Isotherms at *Ra* = 10^3^, (**left**: Present; **Right**: De Vahl Davis 1983) [46]. (**b**) Isotherms at *Ra* = 10^5^ (**left**: Present; **Right**: De Vahl Davis 1983) [46].

The computational mesh chosen after the grid refinement study is presented in Figure 2. Further, to confirm the accuracy of the computational data, the mean *Nu* of this research for various values of *Ra* when *Pr* = 0.71, ϕ1 = 0, ϕ2 = 0, ϕ3 = 0, and *m* = 0 are compared with [45,46] in Figure 3. Also Figure 4 presents an excellent agreement between the isotherms of the current study with [46]. This authenticates the validity of the present model throughout this research work.

## 4. Results and Discussion

In this analysis, we kept *Ra* = 1000, *Pr* = 6.2, CuO nanoparticles volume fraction ϕ1 = 0.04, Ag nanoparticles volume fraction ϕ2 = 0.04, Al_2_O_3_ nanoparticles volume fraction ϕ3 = 0.04, *m* = 0 (constant heating), and *m* = 1 (linear heating) fixed throughout the study unless otherwise specified except for the concerned parameter under analysis.

**Figure 5:**
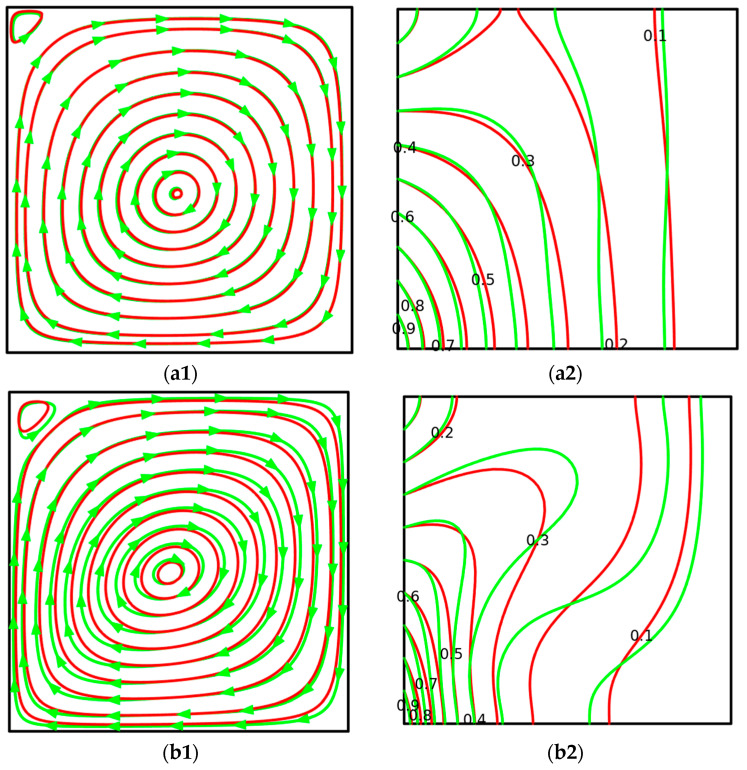

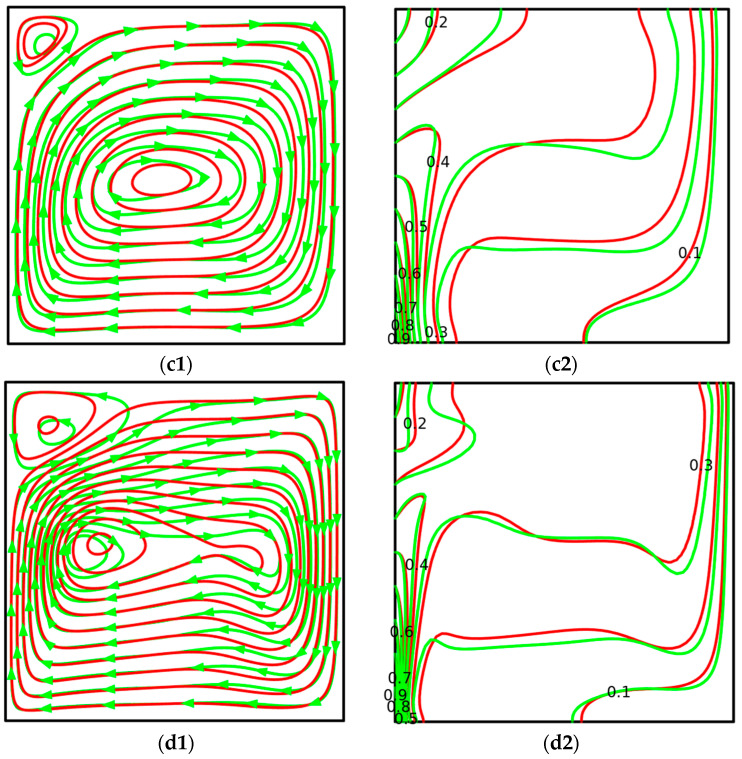
Comparison of streamlines and isotherms between ternary hybrid nanofluid (red lines) and pure fluid (green lines) at various Rayleigh numbers for *m* = 1 (linear heating). (**a1**) Streamlines at *Ra* = 10^3^. (**a2**) Isotherms at *Ra* = 10^3^. (**b1**) Streamlines at *Ra* = 10^4^. (**b2**) Isotherms at *Ra* = 10^4^. (**c1**) Streamlines at *Ra* = 10^5^. (**c2**) Isotherms at *Ra* = 10^5^. (**d1**) Streamlines at *Ra* = 10^6^. (**d2**) Isotherms at *Ra* = 10^6^.

Figure 5 shows streamlines and isotherms within the enclosure for various *Ra* and the working liquid. For the considered case, the temperature of the left vertical border changes from 1.0 at the bottom wall to 0.0 at the upper one. Taking into account this non-uniform warming of the left vertical border, a secondary recirculation can be found in the upper part close to the left corner, while a global circulation is placed in the central part. The appearance of the weak eddy in the upper corner can be explained by the formation of the low wall temperature in this part while the temperature in the major convective cell is high, and as a result, one can find a formation of counter-clockwise circulation in this part. These two cells’ circulation structure is formed within the enclosure regardless of the Rayleigh numbers. For *Ra* = 10^3^, one can find a formation of streamlines like concentric circles and isotherms, illustrating propagation of a high temperature from the lower left corner where a high temperature is maintained. Moreover, the heat transfer mode is heat conduction, and the propagation rate of heat along the horizontal direction is low compared to the vertical direction due to the non-uniform wall temperature. Taking into account such weak circulation, the shape of the considered eddies is circle-like. It should be noted that the shape of the streamlines does not depend on the nanoparticles’ addition due to the low Rayleigh number and domination of heat conduction. Whilst isotherms have some differences, an introduction of nanoparticles increases the effective thermal conductivity, and as a result, heating/cooling of the cavity occurs more intensively in the case of hybrid nanofluid (see differences in red and green lines).

A growth in *Ra* (see Figure 5(b1,b2)) results in the weak modification of the major convective cell structure. One can find a weak elongation of the concentric circles along the cavity’s secondary diagonal due to a reduction of the boundary layer’s thickness near the isothermal walls. The isotherms for this Rayleigh number reflect the appearance of the heat plume close to the left vertical border where an ascending fluid flow is formed. In this case, one can find differences not only in the temperature fields, but also in the flow structure for various working fluids. Thus, the addition of nanoparticles leads to a not-so-essential intensification of the flow, and as a result, the streamlines have very weak elongation, and isotherms reflect the not-so-essential inner stratification zone for the host liquid. Thus, weak modification can be explained by a growth in not only the effective thermal conductivity with nanoparticle concentration but also in the effective viscosity increases. As a result, weak circulation occurs.

Further growth of the buoyancy force characterizes an elongation of the streamlines along the middle horizontal line, and the thermal boundary layer close to the left vertical boundary becomes thinner, while the size of the secondary recirculation increases due to the more essential temperature gradient formed in this zone. In the case of *Ra* = 10^6^ (see Figure 5(d1,d2)), one can find an essential modification of the flow structure with a dislocation of the convective cell close to the left vertical border, a formation of thin dynamic boundary layers near the vertical borders, whilst the central zone is characterized by the formation of a stratified temperature field. Taking into account the formed temperature fields, it is possible to conclude that the growth of the buoyancy force strength increases the average cavity temperature, and the size of the secondary recirculation also rises. Simultaneously, some differences can be found in isolines for pure liquid and hybrid nanofluid due to differences in effective thermal conductivity and dynamic viscosity. The different thicknesses of the boundary layers near isothermal walls characterize the different sizes of the secondary eddy, located in the upper left corner and the major convective cell core.

**Figure 6:**
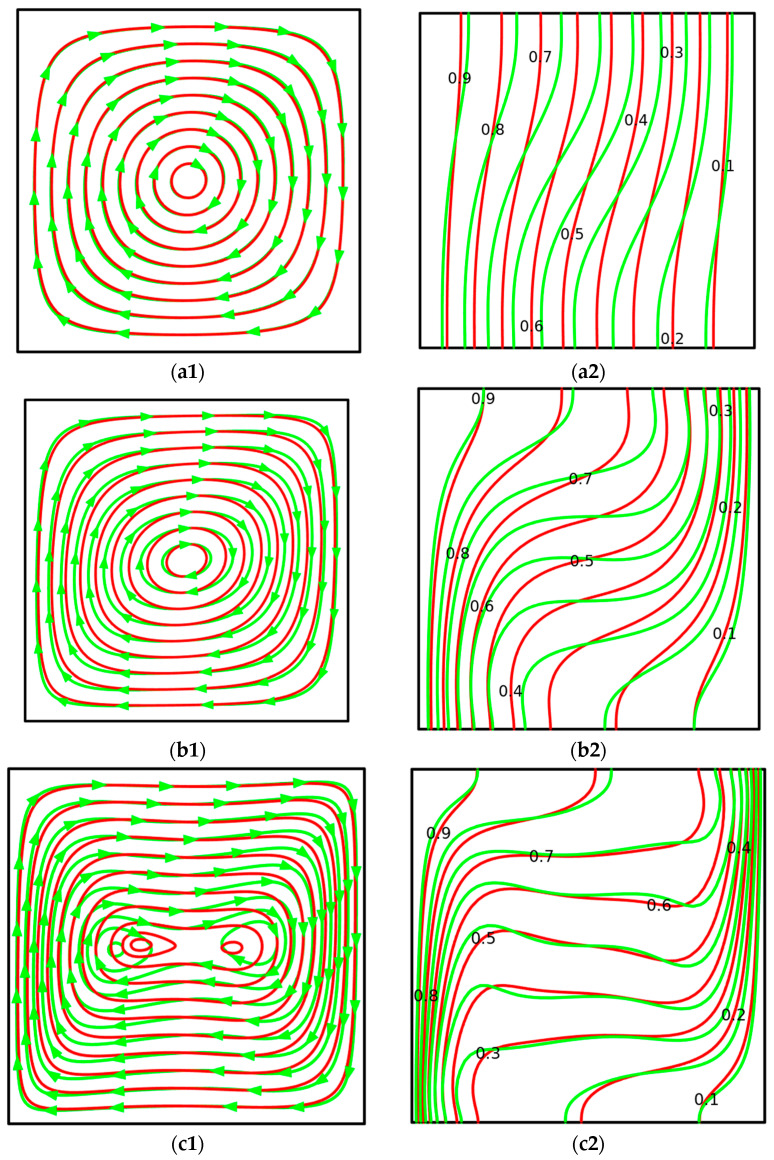

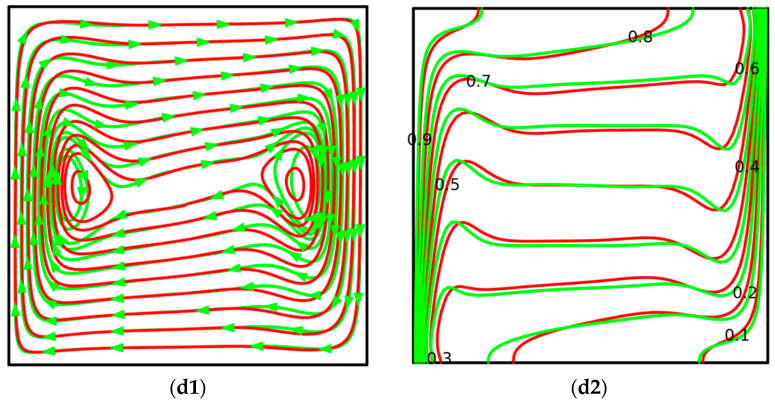
Comparison of isolines of stream function and temperature between ternary hybrid nanofluid (red lines) and pure fluid (green lines) at various Rayleigh numbers for *m* = 0 (constant heating). (**a1**) Streamlines at *Ra* = 10^3^. (**a2**) Isotherms at *Ra* = 10^3^. (**b1**) Streamlines at *Ra* = 10^4^. (**b2**) Isotherms at *Ra* = 10^4^. (**c1**) Streamlines at *Ra* = 10^5^. (**c2**) Isotherms at *Ra* = 10^5^. (**d1**) Streamlines at *Ra* = 10^6^. (**d2**) Isotherms at *Ra* = 10^6^.

For the uniform warming of the left vertical border (*T* = 1), the growth of *Ra* presented in Figure 6 reflects a formation of one clockwise circulation due to warming from the left wall and cooling from the right. These flow structures and temperature patterns are similar to the same fields in the case of a pure fluid without nanoparticles [46].

A raise in *Ra* illustrates a modification of the flow structures from the concentric circles to the circulation with two convective cells placed near the vertical walls, while temperature fields illustrate a strengthening of convective energy transport with the formation of a stratified temperature zone in the central part, which heats from the upper part to the bottom one.

At the same time, the introduction of nanoparticles reflects less intensive circulation and not-so-thin boundary layers near the isothermal vertical walls compared to the pure fluid. The reason for such differences was explained in the case of Figure 5.

**Figure 7:**
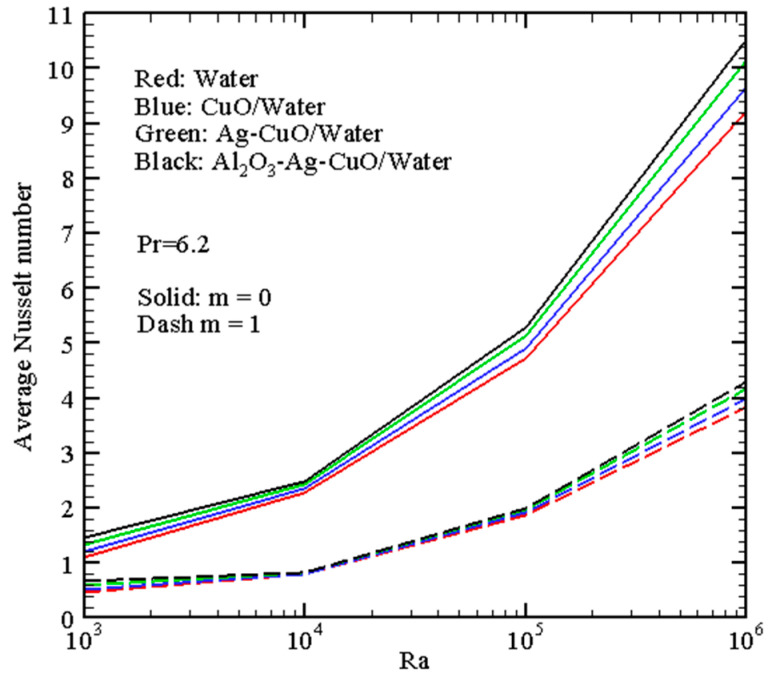
Mean Nusselt number with Rayleigh number.

**Figure 8:**
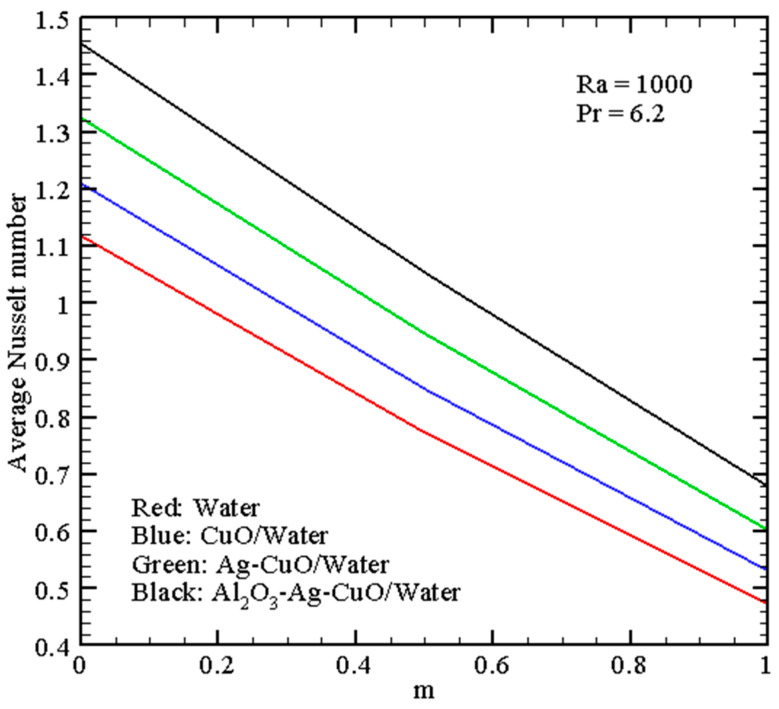
Average Nusselt number with *m*.

**Figure 9:**
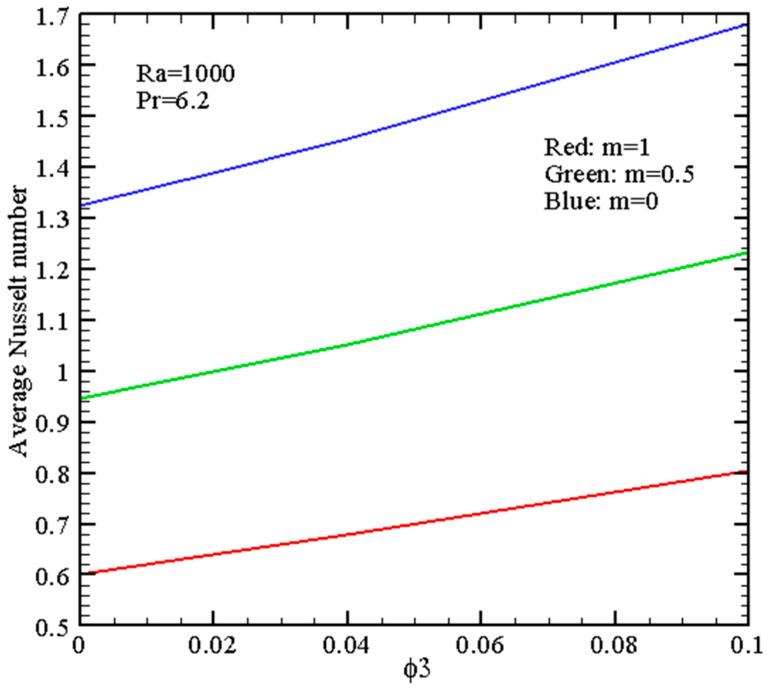
Mean Nusselt number with nanoparticles volume fraction ϕ3.

**Figure 10:**
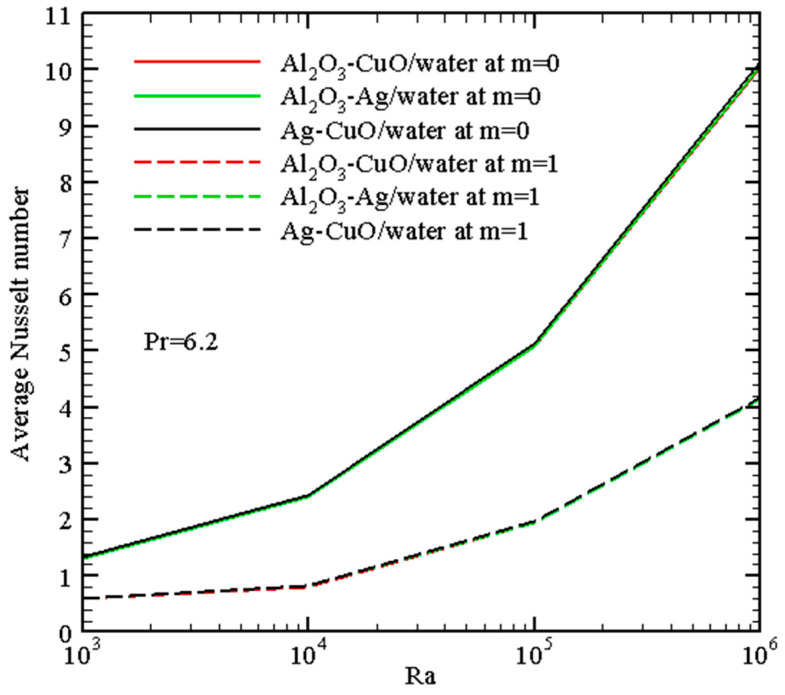
Mean Nusselt number for various hybrid nanofluids with *Ra*.

Figure 7, Figure 8, Figure 9 and Figure 10 demonstrate the mean *Nu* for different governing parameters. Thus, one can find growth of the energy transport strength with *Ra* in Figure 7 due to the formation of more intensive circulation with a larger magnitude of the buoyancy force. An inclusion of nano-additives to the host liquid (water) intensifies the energy transport, and this intensification becomes more essential for high *Ra*. Such an intensification can be explained by the more essential contribution of the thermal conductivity and not the dynamic viscosity because growth of the viscosity characterizes a reduction of the flow intensity.

A transition between the uniform and non-uniform wall temperature profiles characterizes the heat transfer strength augmentation, while the influence of nanoparticles and the Rayleigh number is the same regardless of the character of the left border temperature. The reduction of the average Nusselt number in the case of non-uniform heating compared to the uniform case can be explained by a decrease in the total heat flux from the heated wall.

Figure 8 shows the energy transport enhancement for the nanofluid with the addition of nanoparticles of various kinds. These results characterize the most essential heat transfer enhancement for the ternary nanosuspension. Such an intensification can be explained by the growth of the effective thermal conductivity of the nanosuspension. A rise in the parameter *m* reflects a diminution of the mean *Nu* due to a reduction of the heat energy generated by the warmed left border.

Figure 9 shows the energy transfer augmentation with alumina nano-additives for *Ra* = 10^3^. As mentioned previously, the rise of *m* reflects a decrease in heat transfer strength. Therefore, the major energy transport augmentation can be achieved for high ϕ3 and *m* = 0.

Figure 10 illustrates the energy transport enhancement with the Rayleigh number and parameter *m*. It should be noted that there is no difference between the different considered hybrid nanofluids for the average Nusselt number, namely, Al_2_O_3_-CuO/water, Al_2_O_3_-Ag/water, or Ag-CuO/water. However, the addition of a third kind of nanomaterial for the case of ternary nanosuspension leads to a rise in the energy transport strength (see Figure 8).

Table 5 shows a comparison of the average Nusselt number between pure water and ternary hybrid nanofluid for uniform and non-uniform heating. One can find an essential intensification of the heat transfer with a hybrid nanofluid, and this intensification is more essential for low Rayleigh numbers when heat conduction is the dominant heat transfer mechanism.

## 5. Conclusions

The natural convection of a ternary hybrid nanosuspension in a differential-heated enclosure under non-uniform heating from the left vertical border was studied. The single-phase nanofluid model was used for analysis. The performed analysis showed that

− the addition of a third kind of nanomaterial allows for intensifying the energy transport within the closed chamber. This intensification is more essential in the case of a low Rayleigh number (it achieves 30% for *Ra* = 10^3^) when heat conduction is a dominant heat transfer regime;
− a transition from the uniform heating to non-uniform heating of the left vertical border (from *m* = 0 to *m* = 1) characterizes the heat transfer degradation due to a reduction of the total heat energy output from this warmed border, and this difference becomes more essential with the growth of the Rayleigh number. Thus, for *Ra* = 10^6,^ the average Nusselt number decreases at about 58% with a transfer between *m* = 0 and *m* = 1;
− the presence of a non-uniform heating left vertical wall results in the appearance of secondary recirculation close to the upper left corner. This eddy becomes wider with the Rayleigh number. Moreover, the size of this secondary eddy is large in the case of pure water compared to the nanofluid due to the influence of effective viscosity;
− as a result, the ternary hybrid nanofluids can be used for an intensification of heat transfer in closed chambers, e.g., for cooling of heat-generating elements.

## Figures and Tables

**Table 1:** Physical attributes of Al_2_O_3_-Ag-CuO/H_2_O [23,26].

Attribute	Ternary Nanosuspension (Al_2_O_3_-Ag-CuO/H_2_O)
Density (ρ)	$\rho_{thnf}=\left(1-\phi 3\right)\left\{\left(1-\phi 2\right)\left\{\left(1-\phi 1\right)\rho_{f}+\phi 1\rho_{s1}\right\}+\phi 2\rho_{s2}\right\}+\phi 3\rho_{s3}$
Viscosity (μ)	$\mu_{thnf}=\frac{\mu_{f}}{{\left(1-\phi 1\right)}^{2.5}{\left(1-\phi 2\right)}^{2.5}{\left(1-\phi 3\right)}^{2.5}}$
Thermal volume capacity (ρ*c_p_*)	${\left(\rho c_{P}\right)}_{thnf}=\left(1-\phi 3\right)\left\{\left(1-\phi 2\right)\left[\left(1-\phi 1\right){\left(\rho c_{P}\right)}_{f}+\phi 1{\left(\rho c_{P}\right)}_{s1}\right]+\phi 2\;{\left(\rho c_{P}\right)}_{s2}\right\}+\phi 3{\left(\rho c_{P}\right)}_{s3}$
Volume heat expansion parameter (ρβ)	${\left(\rho \beta \right)}_{thnf}=\left(1-\phi 3\right)\left\{\left(1-\phi 2\right)\left[\left(1-\phi 1\right){\left(\rho \beta \right)}_{f}+\phi 1{\left(\rho \beta \right)}_{s1}\right]+\phi 2\;{\left(\rho \beta \right)}_{s2}\right\}+\phi 3{\left(\rho \beta \right)}_{s3}$
Heat conductivity (κ)	$\kappa_{thnf}=\kappa_{hnf}\frac{\kappa_{s3}+2\kappa_{hnf}-2\;\phi 3\;\left(\kappa_{hnf}-\kappa_{s3}\right)}{\kappa_{s3}+2\kappa_{hnf}+\phi 3\;\left(\kappa_{hnf}-\kappa_{s3}\right)}$, where $\kappa_{hnf}=\kappa_{nf}\frac{\kappa_{s2}+2\kappa_{nf}-2\;\phi 2\;\left(\kappa_{nf}-\kappa_{s2}\right)}{\kappa_{s2}+2\kappa_{nf}+\phi 2\;\left(\kappa_{nf}-\kappa_{s2}\right)}$, and $\kappa_{nf}=\kappa_{f}\frac{\kappa_{s1}+2\kappa_{f}-2\;\phi 1\left(\kappa_{f}-\kappa_{s1}\right)}{\kappa_{s1}+2\kappa_{f}+\phi 1\left(\kappa_{f}-\kappa_{s1}\right)}$

**Table 2:** Physical attributes of host liquid and nano additives [44].

Attributes	H_2_O (*f*)	CuO (s1)	Ag (s2)	Al_2_O_3_ (s3)
ρ (kg/m^3^)	997.1	6320	10,500	3970
*c_p_* (J/kgK)	4179	531.8	235	765
κ (W/mK)	0.613	76.5	429	40
β (1/K)	21 × 10^−5^	1.8 × 10^−5^	1.89 × 10^−5^	0.85 × 10^−5^

**Table 3:** Grid refinement check with *Nu_average_* for *Ra* = 10^3^, *m* = 1, *Pr* = 6.2, ϕ1 = 0.1, ϕ2 = 0.1, ϕ3 = 0.

Elements Number	Nodes Number	Average Nusselt Number
192	145	0.86125
354	250	0.85912
540	361	0.85923
1012	645	0.85877
1510	930	0.85929
2516	1475	0.85912
6536	3731	0.85892
16,946	9374	0.85887
26,352	14,077	0.85888

**Table 4:** Details of optimum grid type (extra fine).

Mesh Information	Mesh Statistics
Number of elements	
Triangles	15,746
Quads	1200
Edge elements	600
Vertex elements	4
Number of Nodes	9374
Average element quality	0.8028

**Table 5:** Comparison of *Nu_average_* between ternary hybrid nanofluid and pure fluid at various Rayleigh numbers.

*m* = 0 (Constant Heating)					*m* = 1 (Linear Heating)			
*Ra*	Base Fluid (Water)	Ternary Hybrid Nanofluid	Increase In Heat Transfer Rate	Percentage of Increase in Heat Transfer Rate	Base Fluid (Water)	Ternary Hybrid Nanofluid	Increase In Heat Transfer Rate	Percentage of Increase in Heat Transfer Rate
10^3^	1.1178	1.4545	0.3367	30.12%	0.47145	0.67839	0.20694	43.89%
10^4^	2.2739	2.4928	0.2189	9.63%	0.79088	0.83589	0.04501	5.69%
10^5^	4.7208	5.2938	0.573	12.14%	1.8651	2.0097	0.1446	7.75%
10^6^	9.2248	10.523	1.2982	14.07%	3.8395	4.3027	0.4632	12.06%

## Data Availability

Data is contained within the article.

## Nomenclature

Ag	Silver
Al_2_O_3_	Aluminium oxide;
*C_p_*	Specific heat at constant pressure (J kg^−1^ K^−1^);
CuO	Copper oxide;
H_2_O	Water
*g*	Acceleration due to gravity (m s^−2^);
*L*	Length of square enclosure (m);
*m* = 0	Constant heating;
*m* = 1	Linear heating;
*Nu_average_*	Average Nusselt number;
*Pr*	Prandtl number;
*Ra*	Rayleigh number;
*T*	Dimensionless temperature;
*T**	Temperature of fluid (K);
*T_c_*	Cooled wall temperature;
*T_h_*	Hot wall temperature;
*v*_1_*	Velocity component in x-direction (m s^−1^);
*v*_1_	Dimensionless velocity component in x-direction;
*v*_2_*	Velocity component in y-direction (m s^−1^);
*v*_2_	Dimensionless velocity component in y-direction;
*x**	Spatial coordinate along horizontal wall (m);
*x*	Dimensionless spatial coordinate along horizontal wall;
*y**	Spatial coordinate along vertical wall (m);
*y*	Dimensionless spatial coordinate along vertical wall;
β	Volumetric thermal expansion coefficient (K^−1^);
κ	Thermal conductivity (J m^−1^ K^−1^);
μ	Dynamic viscosity (Pa s);
ν	Kinematic viscosity (m^2^ s^−1^);
ρ	Density (kg m^−3^);
ϕ1	CuO nanoparticles volume fraction;
ϕ2	Ag nanoparticles volume fraction;
ϕ3	Al_2_O_3_ nanoparticles volume fraction;
**Subscripts**	
*F*	Base fluid;
*hnf*	Hybrid nanofluid;
*nf*	Nanofluid;
*s*1	Solid nanoparticles of CuO;
*s*2	Solid nanoparticles of Ag;
*s*3	Solid nanoparticles of Al_2_O_3_
*thnf*	Ternary hybrid nanofluid
